# Supplementary material for: An Anti-Invasive Role for Mdmx through the RhoA GTPase under the Control of the NEDD8 Pathway
Source: Cells. 2024 Sep 28;13(19):1625. doi: 10.3390/cells13191625 (PMC11475522; doi:10.3390/cells13191625)
Supplement: Supplementary file 1 [file cells-13-01625-s001.zip › cells-3174289-supplementary.pdf]

S1

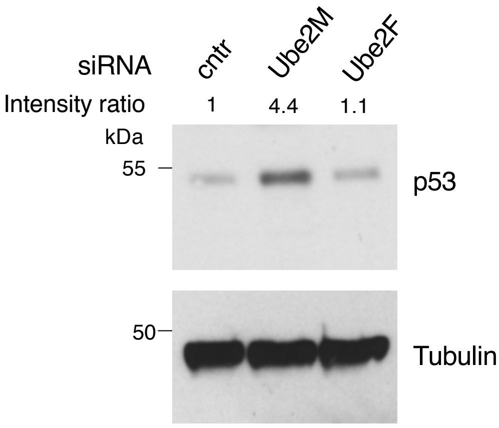

Supplementary Figure S1, related to Figure 1. Extracts from the experiment performed in Fig. 1B were used for western blot analysis using anti-p53 antibodies (DO-1) and anti-tubulin as loading marker. Intensity of p53 bands was normalized against tubulin signal and presented as the ratio for each condition using the cntr as reference.

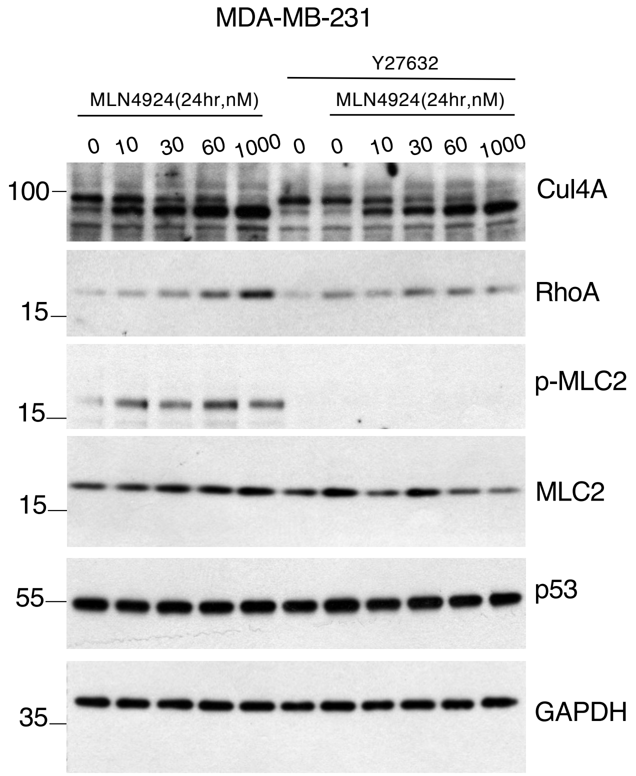

Supplementary Figure S2, related to Figure 3. Experiment performed as in Figure 3E, in MDA-MB-231 cells.

S3

A

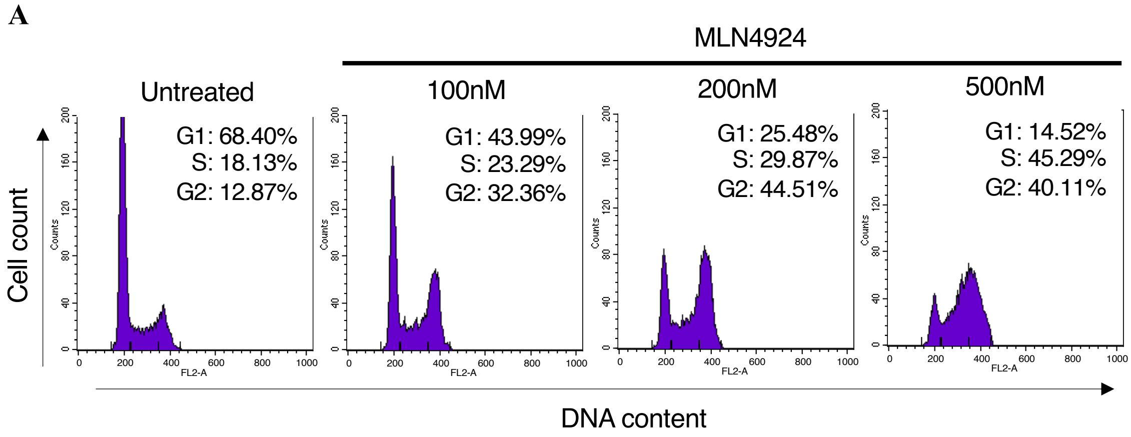

B

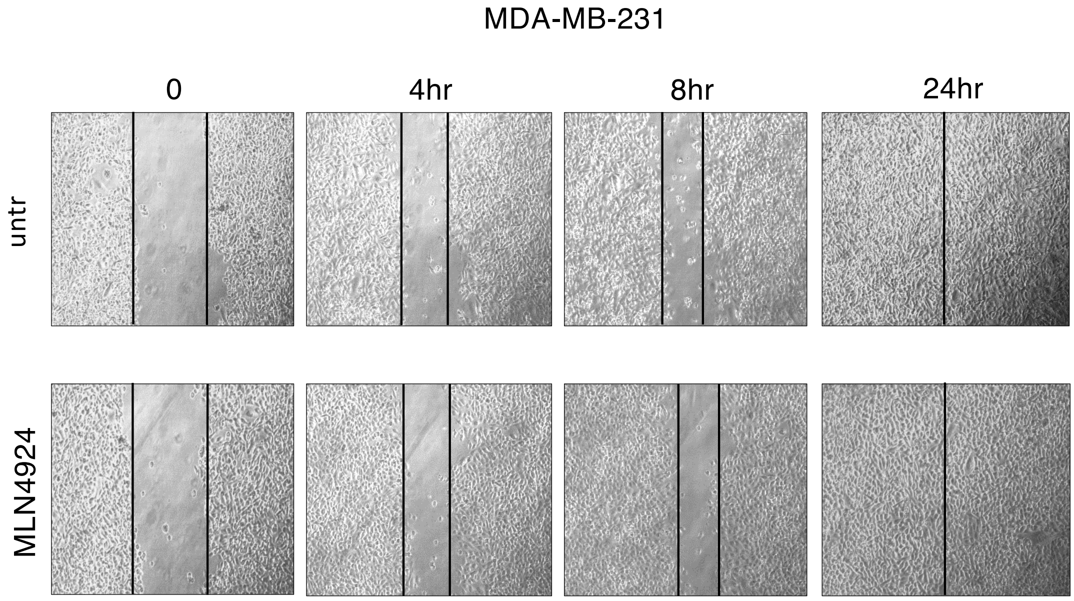

Supplementary Figure S3, related to Figure 7. A. Cell cycle analysis of A375 cells treated with the indicated concentration of MLN4924 for 24hr. **B.** Untreated (untr-DMSO) or MLN4924 treated (100nM) MDA-MB-231 cells were used in a 2D migration assay as described in Materials and Methods. Black lines indicate the gap between cells filled over the indicated period of time.
